# Heteronanotubes: Challenges and Opportunities

**DOI:** 10.1002/smsc.202000039

**Published:** 2021-01-22

**Authors:** Rong Xiang, Shigeo Maruyama

**Affiliations:** ^1^ Department of Mechanical Engineering The University of Tokyo Tokyo 113-8656 Japan

**Keywords:** electronic devices, heteronanotubes, one-dimensional van der Waals heterostructures, syntheses

## Abstract

The experimental synthesis of 1D van der Waals heterostructures, a class of materials in which different crystallized nanotubes are coaxially nested, was recently demonstrated. In this perspective, the current challenges in the chemical vapor deposition synthesis of this new structure are outlined, and the visions on its potential applications are presented. A particular emphasis is given to electronic devices that are built after modulating different shell–shell combinations in these heteronanotubes.

## Graphical abstract

The concept of van der Waals heterostructure was previously only demonstrated in 2D atomic layers but recently extended to 1D nanotube materials. This perspective summarizes the current challenges in the chemical vapor deposition synthesis of these coaxial and tubular heterostructures, i.e., heteronanotubes, and also discusses their possible potentials as building blocks of electronic devices.

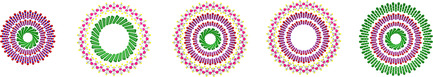

## 1 Brief History

The first observation of a heteronanotube, as far as we are aware of, was reported by Suenaga et al.^[^ ^1^ ^]^ not too late after the discovery of carbon nanotubes (CNTs). Nanoparticles and nanotubes with well‐separated multilayers of boron nitride (BN) and carbon were found in the soot formed by arcing a hafnium diboride (HfB_2_) rod with graphite in nitrogen atmosphere.

Later, some efforts were made to produce such heteroatomic nanotubes in a more intentional and controlled manner. One approach utilized the inner nanospace of a nanotube. Researchers loaded BN precursors into single‐walled CNTs (SWCNTs)^[^ ^2^ ^]^ or reversely, encapsulated fullerene into a BN nanotube (BNNT).^[^ ^3^, ^4^ ^]^ After annealing the structure at high temperatures or by an e‐beam, inner materials transformed into tubular structures due to the confinement of the outer nanotube. By this smart “inner approach”, coaxial structures were formed at local areas of the starting nanotube, although long and continuous heterostructures are difficult to obtain.

One other approach took the outer surface of a CNT as a template (or say a curved substrate) to support the growth of additional layers. This “outer approach” has potential for efficient mass diffusion and continuous production. However, without nanospace confinement, previous attempts suggested that the additional materials are either amorphous or poorly crystalized, and in most cases form 3D bulk crystals.^[^ ^5^, ^6^, ^7^, ^8^, ^9^ ^]^ It seemed not feasible to arrange atomic layers following the circumference of the template into a perfect tubular structure.

**Figure 1:**
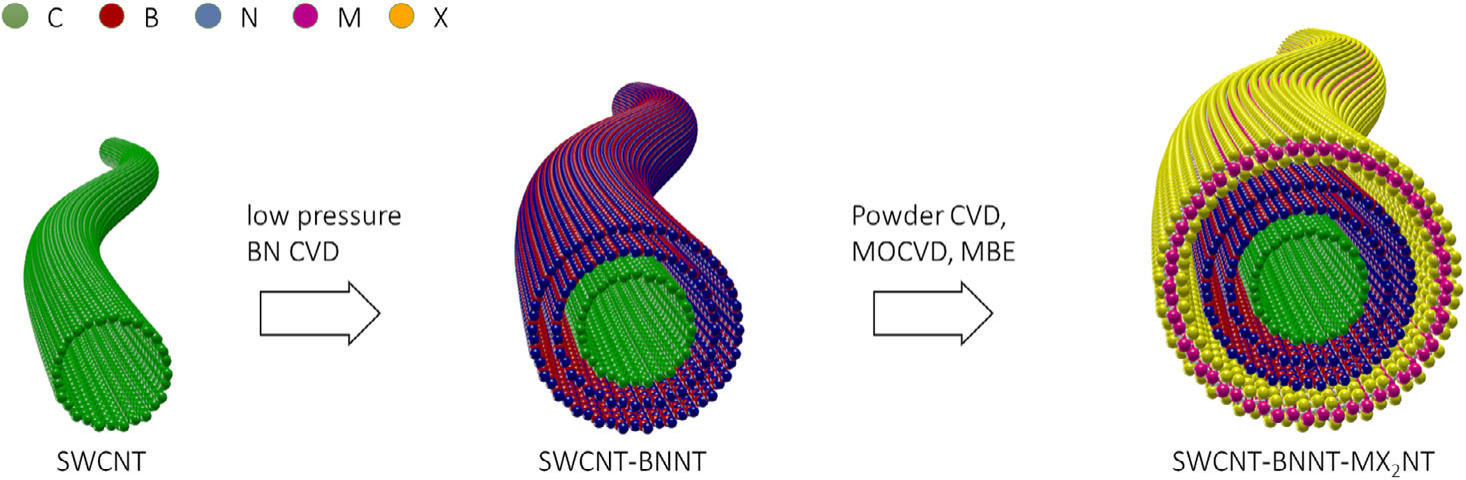
A schematic showing the synthetic approach of a SWCNT–BNNT–MX_2_NT 1D vdW heterostructure, where MX_2_ represented MoS_2_ in our previous report,^[^ ^10^ ^]^ but a similar process can be extended to other TMDC nanotubes.

Earlier this year, we demonstrated the chemical vapor deposition (CVD) synthesis of SWCNT–BNNT and SWCNT–MoS_2_NT heterostructures.^[^ ^10^ ^]^ A ternary SWCNT–BNNT–MoS_2_NT with the diameter of 5 nm was also fabricated by two sequential CVDs (**Figure** **1**). In these structures, different nanotubes are coaxially nested and all shells are single crystals, as demonstrated by transmission electron microscope (TEM), scanning TEM (STEM), electron energy loss spectroscopy (EELS), and electron diffraction characterizations. We noticed that cleanliness of the reaction is key for the growth of perfect outer crystals. This includes the requirement of an ultraclean surface for the starting SWCNT, as well as the requirement of a clean atmosphere within the CVD chamber. In particular, unintentional impurities (e.g., carbon, SiO_2_, or metal atoms) should be minimized, and this is achieved in our case using a low‐pressure CVD chamber. The high degree of crystallization, the moderately long single‐crystal domain, and shell‐by‐shell synthesis manner, convinced us that this structure can be named as 1D van der Waals (1D vdW) heterostructures, heteronanotubes in short.

## 2 Challenges in Synthesis

The growth of 1D vdW heteronanotubes followed an open‐end model, meaning that precursors (molecule, radicals, or atoms) were added to the edge of the outer BN or MoS_2_ nanotube and extended the length of the outer tube. This model is unconventional in the growth of 1D materials.

In most of the previous synthesis processes of CNTs for example, a nanosized catalyst metal particle was needed, and carbon atoms were incorporated into the nanotube through the catalyst. Open‐end growth, also called CNT cloning, was reported only in very few cases.^[^ ^11^, ^12^, ^13^ ^]^ CNT cloning is truly desired for chirality‐specific growth; however, it has been under debate partially because of the extremely low yield and poor reproducibility. It has been demonstrated that some CNTs can extend at open end (clonable) but some cannot even if additional carbon sources are provided. This is probably because edges of these unclonable CNTs are closed, so it is difficult to add new carbon atoms. In the case of heteronanotube growth, thanks to the existence of an inner SWCNT template, outer nanotubes can always keep their edges open. These open edges and atomic steps were clearly visualized by TEM, and the extension of outer nanotubes can be observed by checking the same single heteronanotube before and after a BN CVD.

Although the open‐end growth mechanism is evidenced and straightforward, there are still currently many challenges for our synthesis process, and one of the first to overcome is how to obtain an ultralong heteronanotube.

Because of the absence of a catalyst, the formation of an outer nanotube is extremely slow. The rate of conventional CNT growth from a catalytic metal particle is usually in the range of 1–1000 μm min^−1^.^[^ ^14^ ^]^ In our catalytic‐free process, however, the length of BNNT after a typical 1 hr CVD is around from a few tens of nm to a few hundreds of nm, giving an average growth rate below 10 nm/min. This value is 2–5 orders of magnitude lower than catalytic growth of a CNT. (We also grew 2D BN directly on graphene in a similar condition and confirmed a consistently low growth rate.^[^ ^15^ ^]^) Besides the absence of a catalyst, another reason may be also responsible for this slow growth. Probably, the feeding of precursors to the growth of outer nanotubes is from the gas phase (those that directly collided and interacted with dangling bonds at a tube end) other than via the SWCNT surface. This speculation is partly supported by our TEM observation that the region on SWCNT surface near the open end is usually clean. If precursor molecules or segments (likely to be monomeric aminoborane BH_2_NH_2_ because of its high activity) can be efficiently absorbed onto the surface of a SWCNT and then diffuse along the SWCNT to the open end,^[^ ^16^ ^]^ these intermediate precursors should be identifiable by our cutting‐edge TEMs. Overall, such a low efficiency, in this catalytic‐free and probably gas‐feeding‐dominant process, has made the preparation of ultralong heteronanotubes technically challenging. Having a long and perfect heteronanotube is an essential requirement for many studies (e.g., optical characterizations typically use an excitation beam of micrometers or larger in diameter), but our current sample still cannot be used (at least not routinely used) for these purposes.

However, building long MoS_2_ based heterostructures may be more efficient. In the 2D case, even without a catalyst, the sub‐mm‐large single‐crystal domain of MoS_2_ can be obtained on SiO_2_, graphene, or 2D hBN after reasonably long‐time CVD. Therefore, it could be more feasible to build a long MoS_2_ nanotube, if one can start from a suspended CNT or BNNT and perform MoS_2_ CVD on this nanotube. CNT is an easier option because several approaches are already available to prepare mm‐long and suspended CNTs over a Si slit or pillars. BNNT will be preferred when studying optical properties of the outer MoS_2_ nanotube as it is a transparent insulator. Even though there could be some differences between 1D and 2D growth, we optimistically expect that MoS_2_‐based ultralong heterostructures may be achieved in the near future.

The second challenge we face is mass production. Being able to obtain and provide samples in large quantity is always one key step toward the quick development of a new material.^[^ ^17^ ^]^ Our current protocol for synthesizing BNNTs requires a vacuum chamber and a reaction temperature higher than 1000 °C, both of which are unfavorable for a scale‐up synthesis. The need of a vacuum system may not be indispensable as long as an atmospheric pressure reactor can provide the same level of precursor concentration and cleanness; reducing the reaction temperature while maintaining the efficiency and crystallization is more difficult at this stage, but it may be achieved if a proper way can be found to introduce catalytic atoms into the process.

If a low‐temperature and atmospheric‐pressure process can be developed, the yield of heterostructure will mostly depend on the outer surface of the starting SWCNT, i.e., the effective amount of SWCNT. We say effective amount because we are aware that well‐isolated SWCNTs are needed to form an ideal coaxial structure. Any bundles of SWCNT will be not effective as the coating will occur over the entire bundle. From this aspect, as‐obtained commercial SWCNTs, though nowadays available in large quantities, may not be the best starting material for the synthesis of perfect heterostructures due to the high percentage of bundles. As an alternative, forest‐like vertically aligned arrays may be a good compromise to produce heterostructures in a moderate amount and quality, as there are more individual isolated SWNTs inside these SWCNT forests. The ultimate strategy may be synthesizing SWCNTs and heteronanotubes sequentially in connected reactors. A floating catalytic process may be possible as highly isolated SWCNTs are formed in the gas phase and can be introduced into a secondary reaction continuously. Again, a decently high coating rate is needed otherwise the short residence time in a gas‐phase process would not be enough to guarantee an efficient formation of outer nanotubes.

Another, probably the ultimate, challenge is the synthesis of a heteronanotube with precisely defined inner and outer atomic arrangements, that is, the chirality pair in a heteronanotube. In the simplest example of a double‐walled CNT, a historical study and a recent investigation both suggested no noticeable chirality correlation between inner and outer walls.^[^ ^18^, ^19^ ^]^ In general, simple AB stacking preferred in 2D is not possible for double‐walled CNTs nor SWCNT–BNNT except for very specific chirality pairs.^[^ ^20^ ^]^ Double‐walled CNTs showed peculiar Moire superlattices only for specific pairs of chiralities.^[^ ^21^ ^]^ Therefore, it might be interesting to fabricate 1D heterostructures on the template of a chirality‐specific SWCNT, which could be obtained by recent sorting techniques.^[^ ^22^ ^]^ The effective removal of the surfactant will be needed in these experiments, but the controlled fabrication of heteronanotubes on chirality‐defined SWCNTs will be highly preferred for both fundamental studies and device applications.

## 3 Opportunities for Electronic Devices

Due to the freedom in altering shell–shell combination, and the large number of nanotubes that may be synthesized following similar strategies to MoS_2_ growth,^[^ ^23^ ^]^ potential applications of 1D vdW heterostructures can be vast. In this section, we briefly discuss our current understandings on different configurations of electronic and optoelectronic devices that may be built using 1D vdW heterostructures. Some are possible with the current synthetic capability but some others are still not practical without the further development/optimization of material synthesis.

**Figure 2:**
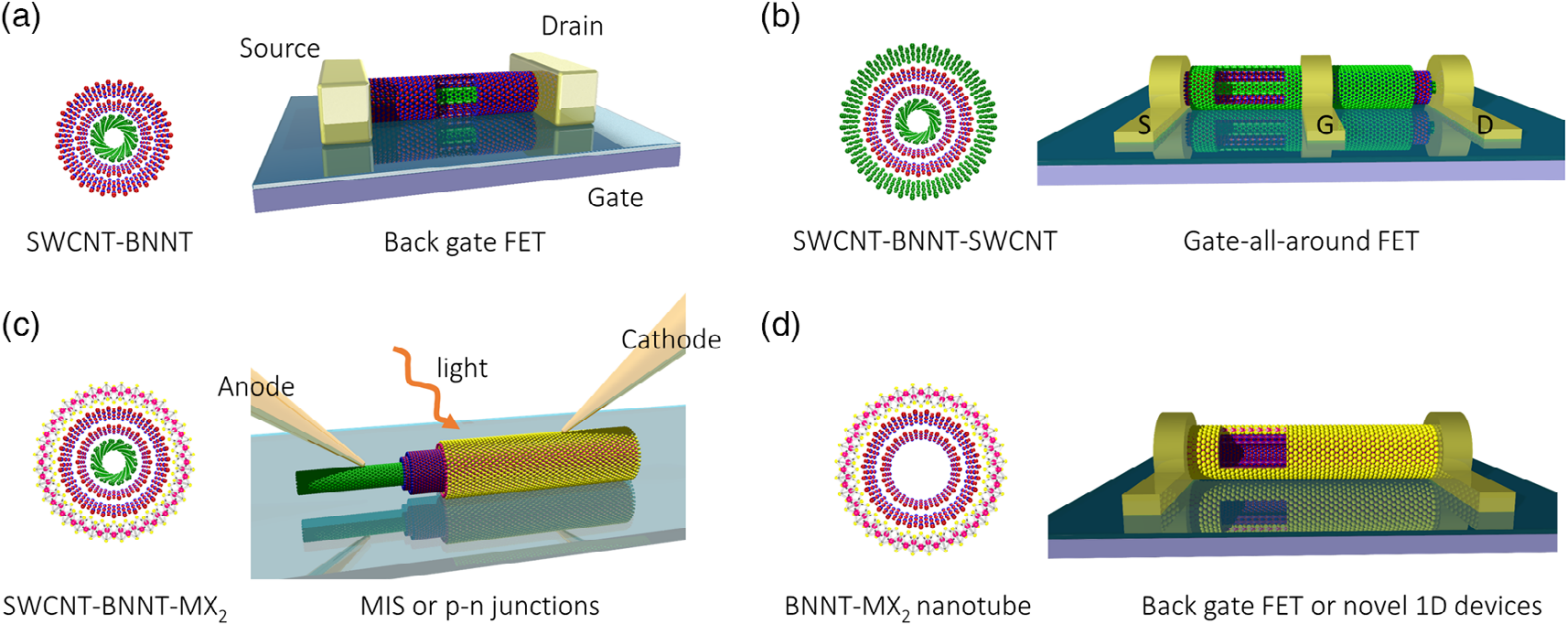
Various electronic and optoelectronic devices that may be built using 1D vdW heterostructures with different shell–shell combinations. a) A back‐gate SWCNT FET using SWCNT–BNNT, b) a “gate‐all‐around” SWCNT FET using SWCNT–BNNT–SWCNT, c) MIS or p–n photo‐detecting/emitting device using SWCNT–BNNT–MX_2_NT, and d) a back‐gate MX_2_NT FET or novel 1D device using BNNT–MX_2_NT.

The first and simplest geometry is to use SWCNT as the channel of a field‐effect transistor (FET) and BNNT as protecting layer, as already demonstrated in our previous report (**Figure** **2**a).^[^ ^10^ ^]^ A similar concept was proven in 2D hBN sandwiched transition metal dichalcogenide (TMDC) devices. In SWCNT–BNNT heteronanotubes, as the channel is completely isolated by BNNTs, we may expect a high‐mobility, less‐hysteresis, smaller environmental effect and higher thermal stability for the SWCNT FET. A simple alteration to the material may achieve a “gate‐all‐around” FET, if a conductive SWCNT or few‐walled CNT can be formed onto the SWCNT–BNNT heteronanotube (Figure 2b). In such a ternary structure, BNNTs serve as the dielectric layer, so probably slightly thicker layers, say four layers, will be needed to prevent the leak from the outer‐most gate.

A second and also straightforward configuration is to use a SWCNT–BNNT–MX_2_ ternary heteronanotube as an optoelectronic device. If the starting SWCNT template is metallic, a SWCNT–BNNT–MoS_2_ heteronanotube is a coaxial metal–insulator–semiconductor (MIS) junction, which may be used as a light‐detecting or emitting device (Figure 2c). In 1D geometry, some interesting features, such as a strong polarization dependence, could be observed. If the starting SWCNT is semiconducting, the resulting structure may form a p–n diode and be used as a solar cell, similar to what was previously demonstrated in silicon nanowire heterojunctions,^[^ ^24^ ^]^ except that the diameter in this case can be one order smaller. However, the bandgap of a SWCNT strongly depends on its helicity so a careful matching of the outermost semiconducting layer is needed

A third configuration can use primarily the property of outer TMDC nanotubes. The current library of 2D TMDC contains semiconductors, superconductors, topological insulators, etc., so there is plenty of room for investigating the unique feature of 1D TMDC materials. One recent discovery is that the 1D WS_2_ nanotube with the diameter of 100 nm can have a giant bulk photovoltaic effect, which is attributed to centrosymmetry breaking.^[^ ^25^ ^]^ In heteronanotubes, sub‐10 nm TMDC nanotubes can be obtained and should have different behaviors from planar 2D crystals. For such applications using the intrinsic properties of outer TMDC nanotubes, we need to avoid a SWCNT core, which can be realized by removing SWCNT after the formation of BNNT. The obtained BNNT may be used for the growth of TMDC nanotubes (Figure 2d).

Very likely, applications other than electronic devices can be also experimentally demonstrated. For example, 1D MWCNT–MoS_2_ and MWCNT–COF heterostructures were recently used in electrochemical reactions and demonstrated decently high performances.^[^ ^26^, ^27^ ^]^ Also, besides TMDC, if other candidate materials in the current 2D library can be also rolled into a heteronanotube, more functions may be realized for these 1D heterostructures. In particular, chemically hetero 2D bilayers have been recently predicted to have superfluid transport that may result in an enhanced Josephson‐like tunneling, dissipation‐less charge counterflow.^[^ ^28^ ^]^ Heteronanotubes in 1D form may also serve as another platform that can host these new physics. In these studies, the curvature‐induced effect, e.g. flexoelectricity, that has been recently calculated in SWCNT‐based nested structures, is expected to cause additional band offsetting, possibly making a straddling (type I) alignment to staggered (type II).^[^ ^29^ ^]^ But along with that, we may still need a lot of effort to improve the synthetic strategies to make these fundamental physical properties accessible.^[^ ^30^, ^31^ ^]^ At this stage, we are still unsure how long this journey may take and where it is finally leading us, but at least it is certain that the door has now opened.^[^ ^32^ ^]^

## Conflict of Interest

The authors declare no conflict of interest.
